# Detection of Sentinel Lymph Nodes in Gynecologic Tumours by Planar Scintigraphy and SPECT/CT

**DOI:** 10.4274/Mirt.236

**Published:** 2012-08-01

**Authors:** Otakar Kraft, Martin Havel

**Affiliations:** 1 University Hospital, Clinic of Nuclear Medicine, Ostrava, Poruba, Czech Republic; 2 University of Ostrava, Faculty of Medicine, Ostrava, Czech Republic

**Keywords:** Sentinel lymph node biopsy, gynecologic neoplasms, scintigraphy, gamma camera Imaging, SPECT, tomography, X-Ray computed

## Abstract

**Objective:** Assess the role of planar lymphoscintigraphy and fusion imaging of SPECT/CT in sentinel lymph node (SLN) detection in patients with gynecologic tumours.

**Material and Methods:** Planar scintigraphy and hybrid modality SPECT/CT were performed in 64 consecutive women with gynecologic tumours (mean age 53.6 with range 30-77 years): 36 pts with cervical cancer (Group A), 21 pts with endometrial cancer (Group B), 7 pts with vulvar carcinoma (Group C). Planar and SPECT/CT images were interpreted separately by two nuclear medicine physicians. Efficacy of these two techniques to image SLN were compared.

**Results:** Planar scintigraphy did not image SLN in 7 patients (10.9%), SPECT/CT was negative in 4 patients (6.3%). In 35 (54.7%) patients the number of SLNs captured on SPECT/CT was higher than on planar imaging. Differences in detection of SLN between planar and SPECT/CT imaging in the group of all 64 patients are statistically significant (p<0.05). Three foci of uptake (1.7% from totally visible 177 foci on planar images) in 2 patients interpreted on planar images as hot LNs were found to be false positive non-nodal sites of uptake when further assessed on SPECT/CT. SPECT/CT showed the exact anatomical location of all visualised sentinel nodes.

**Conclusion**: In some patients with gynecologic cancers SPECT/CT improves detection of sentinel lymph nodes. It can image nodes not visible on planar scintigrams, exclude false positive uptake and exactly localise pelvic and paraaortal SLNs. It improves anatomic localization of SLNs.

**Conflict of interest:**None declared.

## INTRODUCTION

SLN has been incorporated in the routine management of various solid tumor types (1,2,3). Lymph node status in gynecologic tumours remains a most important prognostic factor for recurrence and survival and a major decision criterion for adjuvant therapy (4,5). Despite improvements in imaging techniques, pre-operative assessment of pelvic and paraaortic lymph node status remains difficult (6). Routine pelvic lymphadenectomy and surgical staging are associated with increased risks of complications and morbidity (7). Incidence of nodal metastasis is low in the patient with a low-grade gynecologic tumour (8) with good prognosis (9). The use of lymphatic mapping and SLN identification and biopsy in these patients may help reduce the morbidity of surgery. 

SLN biopsy is the most accurate and the only reliable method for nodal staging which can diagnose microscopic tumour spread to the regional lymph nodes. Minimal invasive SLN biopsy can replace lymphadenectomy for staging. Planar lymphoscintigraphic imaging is an important element in lymphatic mapping but interpretation of planar lymphoscintigrams is hindered by the absence of anatomical landmarks in the scintigraphy image (10). 

In many tumours, lymph node staging is performed using various nuclear medicine procedures, especially sentinel lymph node (SLN) biopsy. SLN biopsy has an established role in malignant melanoma and breast cancer (3,11). A group of patients who might benefit from a SLN biopsy are those with cancer of the uterine cervix and other gynecological malignancies.

In gynecologic malignancies, regional lymph node status is a major prognostic factor and a decision criterion for adjuvant therapy (12). Current FIGO staging is unreliable. The reliability of staging can be improved by laparoscopic staging and new imaging techniques such as PET/CT. These techniques still have to be refined (13).

In our study we compare hybrid SPECT/CT and planar lymphoscintigraphy in patients with gynecologic tumours. 

## MATERIALS AND METHODS

**Patient Population**

Planar and hybrid SPECT/low-dose CT lymphoscintigraphy was performed in 64 consecutive women (mean age 53.6 with a range of 30-77 years) with cervical cancer (T1a-T2, 36 pts, mean age 45.8 with a range of 30-71 years, GROUP A), endometrial cancer (21 pts, mean age 63.8 with a range of 43-77 years, GROUP B) and vulvar cancer (7 pts, mean age 63.6 with a range of 40-77 years, GROUP C) with no clinical evidence of lymph node metastases (N0) and no remote metastases (M0). 

**Lymphoscintigraphic Method**

In patients with gynecologic tumours, we performed preoperative lymphoscintigraphy utilizing 99mTc-colloid, activity 40 MBq, on the operation day (one-day protocol). Gynaecologists injected 4 peritumoural injections of colloid around the tumour. Scintigraphy followed 25-60 minutes after injection. 

We have used these 99mTc – colloids: Nanocoll (size of colloid particles to 80 nm-53 pts) and NanoAlbumon (size of particles to 60 nm-11 patients). Choice of radiopharmaceuticals (Rf) was totally random. Type of Rf was not important because we have compared planar scintigraphy and SPECT/CT performed by the same Rf. We previously reported the success of detection of SLNs by means of various Rfs (3,14,15,16) and this is not necessarily the aim of our present study. 

Planar and SPECT/CT lymphoscintigraphy was performed using a hybrid system composed of a dual-head gamma camera with a low-dose CT installed in a gantry (Symbia T2 Siemens).

Planar lymphoscintigraphy was carried out in the anterior and posterior projections focusing on the area of interest. Acquisition time was 10 minutes. If the sentinel lymph node was displayed, a reference mark was placed on the skin corresponding to the position of the SLN visualised by lymphoscintigraphy with the help of the 57Co mark, to facilitate the surgical resection,. 

SPECT/CT images were acquired immediately after planar images. The SPECT/CT system (Symbia T2; Siemens, Erlangen, Germany) consisted of a dual-head variable-angle gamma camera equipped with low-energy high-resolution collimators and a two-slice spiral CT scanner optimized for rapid rotation. SPECT acquisition (matrix 128x128, 60 frames at 25 s per view) was performed using steps of 6^0^. CT scan was a low-dose, noncontrast study (130 kV, 17 mAs, B60s kernel), 5-mm slices were created. The iterative reconstruction (OSEM 3D) was used for generating SPECT slices. Both SPECT images and CT axial slices were fused using an Esoft 2000 application package (Siemens, Erlangen, Germany). Hybrid SPECT/CT images were viewed using two-dimensional orthogonal re-slicing in axial, sagittal and coronal orientation. Maximum intensity projections with a three-dimensional display were generated to localise sentinel nodes in relation to anatomical structures. 

**Figure 1:**
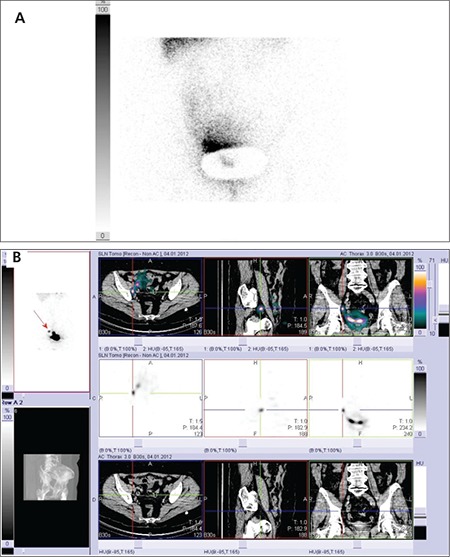
69 year old woman with endometrial cancer. A) In the planar image in anterior projection, only one suspected deposit on the right is observed which is difficult to localize. B) The SPECT/CT fusion image shows one iliac sentinel node in the right

The surgeon is notified of the findings on both planar and SPECT/CT images (Figure 1). An intraoperative hand-held probe (NEO 2000, Neoprobe Corporation Dublin, Ohio, USA; detector: crystal of Cadmium Zinc Telluride; 12 mm collimated angulated probe) is used before incision to identify the site with the highest counts of lymph nodes in the lymphatic basin. A patent blue dye (BLEU PATENTE V 2.5%, Guebert, France) is injected similarly to the earlier colloid injection. 

**Scintigraphic Interpretation**

**Figure 2:**
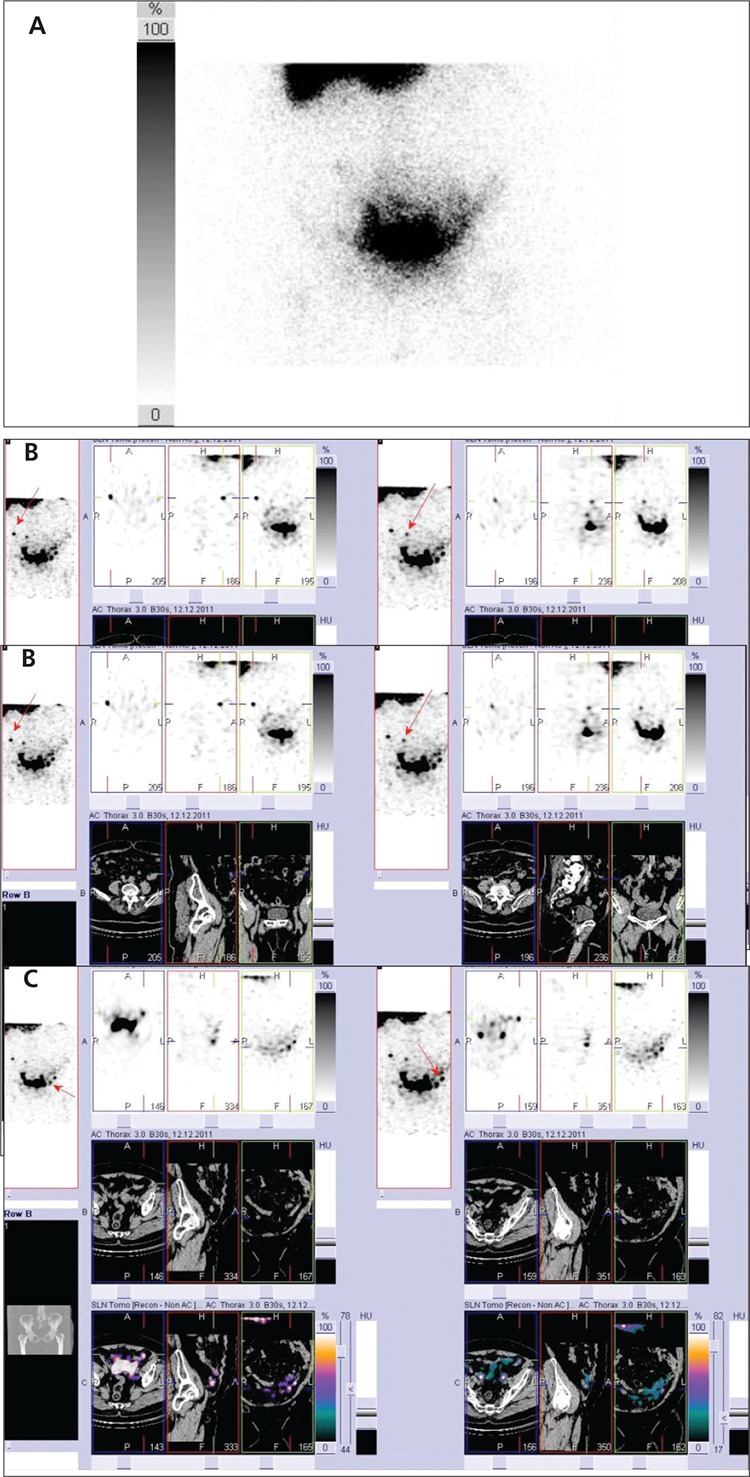
73 year old woman with endometrial cancer. Planar scintigram in anterior projection (A) shows only suspected SLN on the right without possibility to localize it. Fusion of SPECT/CT (B, C) shows two SLNs on the right(B) and two SLNs on the left side (C) of the pelvis

SLN localization was interpreted separately on planar and SPECT/CT images. Image analysis was performed prospectively by two experienced nuclear medicine physicians in consensus reading. In the analysis of the results, fused SPECT/CT image data were concluded to be clinically relevant if it identified SLNs that were missed on planar images, if it excluded SLN suspected on planar images, or if it localized SLNs in addition or in different basins than those suggested by planar images (Figure 2).

**Statistical Test **

Student's paired t-test was used for comparing numbers of detected nodes by both techniques. Values were considered significant when p<0.05. 

## RESULTS

On SPECT/CT images, 240 hot nodes in 60 of the 64 (93.8%) study patients were detected, with a mean of 4.0±2.2 (range 1-11) nodes per patient. SPECT/CT showed the exact anatomical location of all visualised sentinel nodes. There was failure to detect SLNs in the remaining 4 (6.3%) patients. Planar images identified 177 SLNs in 57 (89.1%) women, with a mean of 3.1±1.9 (range 1-9 nodes) per patient. In the remaining 7 (10.9%) patients no SLNs were detected on planar images.

Sixty six lymph nodes in 35 (54.7%) patients were missed on planar images, but identified on SPECT/CT. Three foci of uptake in 2 patients interpreted on planar images as hot LNs were found to be false positive non-nodal sites of uptake when further assessed on SPECT/CT (1.7% from totally visible 177 foci on planar images). In 3 (4.7%) patients, who had negative planar imaging, SPECT/CT visualised lymphatic drainage.

In GROUP A, planar lymphoscintigraphy alone visualized SLN in 32 (88.9%) patients as compared to SPECT/CT imaging that identified SLN in 35 patients (97.2%). The number of SLNs captured on SPECT/CT was higher than on planar imaging in 22 (61.1%) patients.In GROUP B, there were SLN identified in 18 (85.7%) patients on planar images as well as on SPECT/CT, the number of SLNs visualised on SPECT/CT was higher in 10 (47.6%) patients.

In GROUP C, planar lymphoscintigraphy visualized SLN in all 7 patients, same as on SPECT/CT, the number of SLNs viewed on SPECT/CT was higher in 3 (42.9%) patients. 

Differences in detection of SLNs between planar and SPECT/CT imaging in the group of all 64 patients are statistically significant (p<0.05).

Intraoperative SLNs detection (hot and blue positive; only hot; only blue) in all three groups found 254 SLNs. SLN involvement was identified in five SLNs (1.97% of 254 removed SLNs) in 5 patients (7.8% of 64 patients). Four of the five positive SLNs presented a single micrometastatic deposit. One SLN with micrometastasis was detected by SPECT/CT and not by planar lymphoscintigraphy, i.e. only one node identified only on SPECT/CT was positive for tumour. Four metastatic SLNs was detected by means of SPECT/CT and planar scintigraphy. 

## DISCUSSION

The sentinel node (SLN) was defined as the first lymph node draining the primary tumour, ie. the first lymph node that is at risk from metastatic cells (17). The histological status of the SLN has been found to be an indicator representative of the whole lymph node basin. It has turned out to be the strongest predictor for tumour recurrence and survival (18).

Lymphatic mapping has been applied extensively in breast cancer and melanoma patients. Amongst gynecological cancers, the SLN concept has been most accepted for vulvar cancers (19,20). 

A number of researchers have studied SLN detection in vulvar, endometrial and cervical cancer patients (21,22,23,24,25,26). 

The main factors which have influence on the prognosis of gynecologic tumours are: disease stage, type, size and differentiation of tumour. The most important prognostic factor is the state of the lymph nodes. Large part of early stage gynecologic cancers is node-negative. For example, affliction of the lymphatic system in the stage FIGO I of cervical cancers is up to 15%, in the stage FIGO II it is 25.3% (16). In endometrial cancers, the incidence of lymph node metastases is approximately 10% for clinical stage I and occult stage II cancers (27). Radical surgery includes lymph node dissection. This means that the majority of patients derive to no therapeutic benefit from the procedure yet must endure the associated morbidity of lymphadenectomy (28). Complications resulted from extensive radicalism of surgery are lymphocyst formation, lymph drainage blockade with lymphoedema formation of lower extremities, recurrent erysipelas. There is not suitable pre-surgery examination procedure of detection of impacted lymph nodes. SLN biopsy can be feasible in gynecologic cancers and may result in custom-designed treatment strategies with a reduction in morbidity. The most important benefits of the SLN procedure for the patients with cervical cancer are avoidance of over treatment and prevention of morbidity (29). The same is valid and was proven for other gynecologic tumours (30). 

The use of lymphatic mapping and SLN identification in these patients may help reduce the morbidity of surgery without compromising the identification of higher-risk patients who require adjuvant treatment. The SLN protocol of enhanced pathologic evaluation of removed nodes may also provide a much more detailed evaluation of these nodes and the potential for identifying micrometastasis that may have been missed with traditional pathologic evaluation (31). 

In vulvar cancers the difference in morbidity between patients who underwent a detection and dissection of SLN, and patients who underwent radical vulvectomy, including inguinofemoral lymphadenectomy, was clearly shown in the largest observational multicentric study of van der Zee et al. (32). To minimise the risk of false negativity, an experienced multidisciplinary team is the most important factor for successful detection of SLN in early-stage vulvar cancer patients (26). SLN biopsy performed only by an experienced team is a feasible method with high accuracy in patients with early-stage vulvar cancer, with tumour preferably not greater than 3 cm in diameter (26). The usefulness of SLN biopsy in the early stages of cervical cancer with highly negative predictive value was proven (33,34). In gynecologic malignancies, SLN biopsy has been validated in vulvar cancer (19), but its accuracy in cervical cancer is still under evaluation (35). Sensitivity and negative predictive values have to be improved before the concept can be integrated into clinical practice (16). The question remains, whether systematic lymphadenectomy could be omitted at this moment. Several unanswered questions need to be discussed: patient selection criteria; technical aspects, detection methods and learning curve; evaluation per side of detection; and false-negative noninferiority margin (28). Improving and standardisation in all these aspects could reduce false-negative rate, and help to achieve a 5% false-negative rate, which seems acceptable in accordance with other malignancies for which SLN is recognized (36).

Overall results of SLN detection for endometrial and cervical cancer are variable, with higher sensitivity reported with combined techniques – lymphoscintigraphy, probe and blue-dye (23,24,25). In the cervical cancers with the site of cervical injection being close to the sites of SLN basins in the pelvis, the SLN uptake detection by planar lymphoscintigraphy can be difficult due to scatter from the site of injection in cervix.

Accurate visualization of the SLN is required for the best results. Conventional lymphoscintigraphy provides planar images with views in only anterior or lateral projection and poor spatial information on the location of pelvic SLN. In our study, we have done planar imaging only in anterior and posterior projections without lateral images. We firmly believe that it does not limit the comparison of planar versus SPECT imaging. According to our experience lateral images compared with anterior and posterior projections have no role for the detection of other “new” SLNs. It is very important to image lymph nodes in anterior and posterior projections with and without lead shielding located above injection sites. Other situation is in the detection of SLNs in patients with breast cancer and melanoma where lateral planar projections are necessary.

A decade ago, hybrid imaging combining single-photon emission tomography (SPECT) with computed tomography (CT) was introduced for simultaneously acquiring functional and morphological information (28). Hybrid SPECT/CT camera fuses tomographic lymphoscintigrams (physiological information) with anatomical data from CT (37,38). In comparison with traditional single-modality imaging approaches, the dual-modality systems offer unique capabilities in combining data from two imaging modalities in way that simplifies, yet facilitates, image correlation with the goal of revealing useful diagnostic information that is not easily extracted when the imaging studies are performed independently (39). Hybrid SPECT/CT provides better contrast and resolution than planar imaging with possibility to correct attenuation and scatter (40,41). SPECT/CT images provide the topographic landmarks that may further facilitate surgical exploration (38) with improvement of surgical SLNs detection. If only used to correct the radionuclide image for photon attenuation, the CT data can be acquired with a considerably lower statistical quality and coarser spatial resolution than required for diagnostic-quality imaging and therefore can deliver a significantly lower radiation dose than that for a diagnostic CT study (39). Planar nuclear medicine image fusion with CT topograms has been proven feasible and offers a clinically suitable compromise between improved anatomic details and minimally increased radiation dose (10). Hybrid system allows transmission (low-dose CT) and emission (SPECT) scans to be performed without changing the patient´s position, thereby allowing for automatic and correct record of images obtained with two modalities. 

SPECT/CT was introduced in lymphatic mapping with the goal to show more sentinel nodes and to show them more clearly than is possible with conventional lymphoscintigraphy to improve nodal staging (42). Martinez et al. has proved that SPECT/CT accurately detected preoperative SLN topography and enhanced diagnostic sensitivity of SLN imaging, improving surgical approach to patients with cervical cancer staging and diagnostic quality of anatomic landmarks of CT images of SPECT/CT could be further improved by the use of contrast injected CT (28). Planar scintigraphy provides poor visual information on anatomic location of pelvic SLN, and external iliac, internal iliac, obturator, presacral and common iliac nodes are indistinguishable. SPECT/CT allows for easier correlation of areas with physiological variants or abnormal tracer accumulation to anatomical landmarks, enabling to obtain precise preoperative topographic localisation of SLN and to directly guide the surgeon to SLN location for removal. By visualizing SLN location and neighbouring structures by SPECT/CT information, less invasive treatment and thus a reduction of operative time, blood loss, and morbidity is likely to occur (28). SPECT/CT has also been reported to reduce the false-positive interpretation of radiotracer accumulation in the event of contamination or radiotracer in lymphatic vessels (43). One of the limitations of planar imaging in the detection of SLN is its inability to distinguish nodes that are superimposed (44). SPECT/CT lymphoscintigraphy in vaginal melanoma detects a right perirectal SLN that was not detected by planar imaging. This was likely due to the superimposition of inguinal SLN (45). A node close to the injection site can also be masked as the result of strong activity from the injection site (the “shine effect“) (46). The use of SPECT as well as coregistered CT images in vaginal melanoma proves to be useful in detection and localization of SLN not seen on planar imaging alone for use in staging and treatment planning (45). 

The use of routine SPECT/CT imaging in vaginal melanoma for pelvic lymphoscintigraphic studies or as an adjunct tool for localizing SLN in cases that would not be demonstrated with planar imaging alone (45). Preoperative SPECT/CT lymphoscintigraphy is ideal for mapping the unpredicted lymphatic drainage pathways within the complex pelvic anatomy in vulvovaginal melanoma and this technique may also be used in the preoperative workup of other gynecologic malignancies (44) and this novel system added important information to that provided by planar imaging and played a critical role in surgical planning, clinical staging, and subsequent patient management (44). 

The use of SPECT/CT has been described in SLN lymphadenectomy for breast cancer, head and neck cancer, melanoma, prostate cancer, bladder cancer, vulvar, cervical and endometrial cancer - the value of SPECT/CT for SLN identification and localization has been described in several reports (28,31,47,48,49,50,51,52,53,54,55). In special instances, SPECT/CT imaging allows for improved detectability and interpretation of lymphatic drainage. Contamination, nodes close to the injection site, and overweight patients are three noted instances in which SLN identification and localization are better with SPECT/CT than with standard planar methods.

Low radiation dose is added to the scintigraphic mapping by the low-dose CT, ranging from 1.3 mGy at the centre to 5 mGy at the surface of the body (38). The better resolution of SPECT itself, the improved quality of attenuation-corrected SPECT images and the improved anatomical localisation of nodes offered by the three-dimensional data of the SPECT reconstructed planes as well as the anatomical landmarks on CT may have contributed to the better nodal identification by SPECT/CT found in the study of Lerman et al. (56).

Nonvisualization of SLNs is higher in overweight patients with breast cancer (57). Lerman et al. (57) stated that the rate of false-negative planar imaging results for 122 overweight and obese patients was 28%, higher than that for the general study population. The rate of false-negative SPECT/CT results for these 122 patients was also higher than that for the general study population, 11%; however, the latter modality identified hot nodes in 18 additional patients (53%) and had a statistically higher rate of detection of SLNs in overweight patients. The addition of SPECT/CT to the acquisition protocol for lymphoscintigraphy in overweight and obese patients with breast cancer improves the identification of SLNs and avoids false-positive interpretations of sites of nonnodal uptake.

Before the introduction of SPECT/CT, various methods were described to improve the visualization rate of sentinel nodes on planar images. Alterations in the colloid particle concentration, in the amount of radiotracer and in the time of imaging (early versus delayed), a second injection of the radiopharmaceutical, and post-injection massage have all been advocated to enhance the number of visualized SLNs (58,59,60,61). The combination of all these improvements in the technique has led to a high sensitivity of lymphoscintigraphy. Some authors have stated that SPECT/CT, therefore, should only be performed in selected patients, i.e. those with an unusual lymphatic drainage pattern, with planar images that are difficult to interpret or with no visualization on planar images. In these patients, SPECT/CT appears to have additional value (62). Moreover, SPECT/CT provides an anatomical overview in two- and three-dimensional perspectives creating a surgical road-map that cannot be provided by planar images or intraoperative lymphatic mapping techniques. The present study confirms the additional value of SPECT/CT in the anatomical localization of SLNs and underlines its relevance for the surgical approach. SPECT/CT in our opinion, therefore, facilitates surgical exploration in difficult cases and may improve staging. Other investigators have also concluded that additional SPECT/CT after planar lymphoscintigraphy resulted in an improved anatomical localization of SLNs. Especially, SLNs outside the axilla and nodes close to the injection site were easier to identify using SPECT/CT (43,56,62). Neither in the present study nor in studies by other investigators did SPECT/CT miss a sentinel node that was visualized by planar lymphoscintigraphy (42). SPECT/CT was able to accurately bring to light sites of skin contamination with the radiopharmaceutical.

Even if SPECT/CT is not successful, it seems worthwhile to not give up. By careful exploration of the pelvis with the combined use of blue dye, a gamma probe and intraoperative palpation, a fair number of patients can be identified as node-positive and undergo the pelvic clearance they need and others can be spared such a procedure that does not benefit them. 

Ploeg et al. (62) limited the use of SPECT/CT to difficult and unusual cases because they believe planar lymphoscintigraphy is an excellent preoperative mapping technique for most patients. They added nonvisualization as a new indication, because SPECT/CT visualized drainage in patients whose planar images did not reveal a SLN. They believe that the added costs and extra time for SPECT/CT are more justified when the procedure is used for this new indication. SPECT/CT is useful for finding the exact anatomic location of sentinel nodes and in detecting additional sites of drainage. These advantages facilitate surgical exploration and eventually lead to more accurate staging. SPECT/CT may also obviate preoperative skin marking and may replace delayed lateral planar imaging. Whether SPECT/CT should be used on all patients or only for specific indications needs to be studied further (62). The better anatomic definition and improved resolution that characterize SPECT images may overcome limitations of planar images. Localization of hot lymph nodes on SPECT images without anatomic landmarks is not possible. However, it is possible by fusing the SPECT image with the anatomic data obtained by performing low-dose CT at the same setting as with the SPECT acquisition (37). Using SPECT imaging, there was increased sensitivity of SLN detection, with a higher detection rate using SPECT/CT as compared to planar lymphoscintigraphy in all types of gynecologic tumours. The likely reason for false negative result on planar scintigraphy may be the proximity of the nodes to the site of injection: these nodes may be located in bilateral parametrial regions with significant adjacent activity seen in the pelvis secondary to the injection site. Increased background activity from the injection sites could have led to nondetection of the nodes by planar scintigrams in some cases.

Due to the combined imaging techniques of SPECT and CT, it was possible to distinguish nodal uptake from injection site activity. This would again be difficult to detect on planar imaging due to the lack of spatial resolution and corresponding anatomic data. Scattered activity from injection site in general makes detection of these nodes difficult by probe as well as planar imaging. As compared to planar imaging, SPECT was able to more accurately localize various nodal sites in the pelvis. Planar imaging generally cannot accurately distinguish between pelvic nodal sites; in particular, nodes that are closely placed, such as obturator versus external iliac nodes and parametrial nodes, would be difficult to detect. However, detection of such nodes is readily possible with SPECT imaging (31). In this study, nodes were accurately located to the anatomic sites. Accurate anatomic localization of SLN nodes preoperatively can also aid in probe-directed surgery and may reduce operator dependant variation and time. SPECT can accurately localize paraaortic nodes as compared to planar imaging. Overall, multiple nodal sites were detectable in SPECT imaging. SPECT/CT imaging is highly sensitive for SLN detection. It accurately localizes SLN to anatomic locations and improves intraoperative detection of sites. Preoperative SPECT/CT appears to enhance the topographic localization of SLN that may aid the surgeon in localizing SLN against surrounding structures. It is reasonable to consider utilizing SPECT/CT in conjunction with planar imaging when feasible, especially in patients with negative planar imaging, in the hope of enhancing SLN detection and localization. If these data are confirmed, the routine use of SPECT in all patients may potentially have incremental value.

Our study highlights the usefulness of SPECT/CT imaging for sentinel node detection in gynecologic cancers.

## CONCLUSION

The addition of SPECT/CT to planar lymphoscintigraphy may improve the localisation of preoperative draining nodes in patients with vulvar, cervical and endometrial cancers. It may detect SLNs nodes missed by planar imaging, exclude nonnodal false positive sites of uptake and accurately localize pelvic and paraaortic nodes. Further research is warranted to assess the exact value of additional intraoperative detection of SLNs.
